# Supplementary material for: Implementation of a Multidisciplinary Allied Health Optimisation Clinic for Cancer Patients with Complex Needs
Source: J Clin Med. 2020 Jul 30;9(8):2431. doi: 10.3390/jcm9082431 (PMC7465605; doi:10.3390/jcm9082431)
Supplement: Supplementary file 1 [file jcm-09-02431-s001.pdf]

Supplemental Materials:

Figure S1: Phase 1 Model of Care.

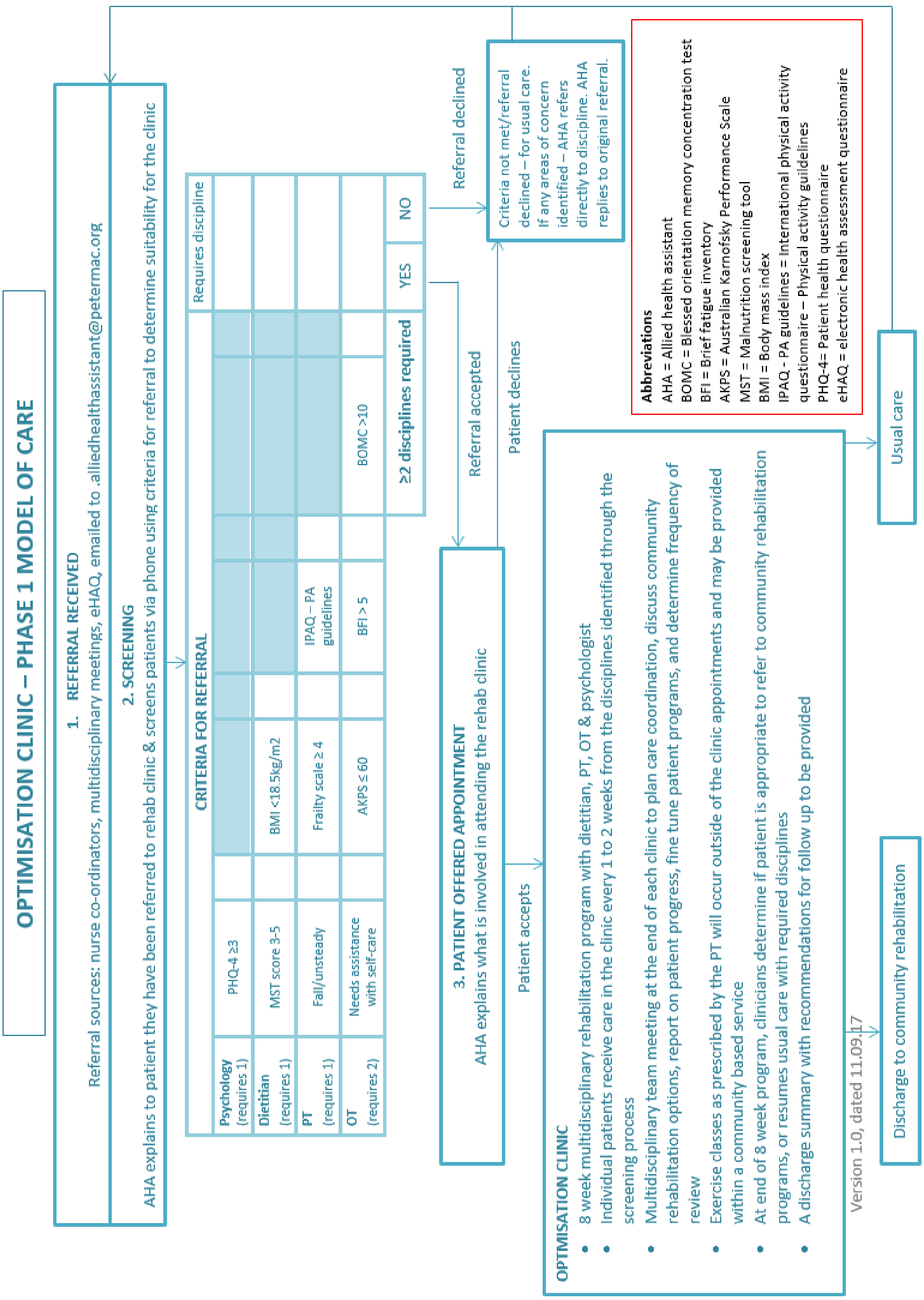

Figure S2: Interview Questions and Prompts.

Focus of the interview: To understand the experience of patients who received treatment in the optimisation clinic, with a focus on patient acceptability of the service.

Interview

You have recently completed treatment in the allied health optimisation clinic. We are interested in hearing your views of the treatment you received in the clinic.

1. Which clinicians did you see in the clinic?
   a. Dietitian, physiotherapist, occupational therapist, psychologist
2. Did you attend the clinic prior to having treatment or at some other time during or after your treatment?
3. Did you attend all clinic appointments? If not, can you please explain why?
4. Can you tell us about your experiences of seeing these clinicians within the one clinic?
5. How did you find the treatment you received in the clinic?
6. How did you feel about the amount of time you spent in the clinic at each visit?
   a. Enough, too much, not enough? (prompt if required)
7. How did you feel about the amount of support provided to you by the clinicians in the clinic?
   a. Enough, too much, not enough? (prompt if required)
8. Were there any specific parts of your treatment in the clinic that you would like to comment on? (Prompt if required – was there anything you found to be beneficial or anything you think could have been left out?)
9. Was there any treatment or education you would have liked to have been provided with in the clinic that you didn't receive?
10. Were you able to do everything that was prescribed by your clinicians in the clinic?
11. If you made changes to your lifestyle as a result of being involved in the clinic, have you managed to continue with these changes? E.g. exercise or activity levels, dietary modifications.
12. Do you have any other comments you would like to make about the clinic or the treatment you received?
